# Cropland connectivity affects genetic divergence of Colorado potato beetle along an invasion front

**DOI:** 10.1111/eva.13140

**Published:** 2020-10-08

**Authors:** Fangyuan Yang, Ning Liu, Michael S. Crossley, Pengcheng Wang, Zhuo Ma, Jianjun Guo, Runzhi Zhang

**Affiliations:** ^1^ Institute of Entomology Guizhou University Guiyang Guizhou China; ^2^ Key Laboratory of Zoological Systematics and Evolution Institute of Zoology Chinese Academy of Sciences Beijing China; ^3^ Department of Entomology University of Georgia Athens GA USA; ^4^ College of Life Science University of Chinese Academy of Sciences Beijing China

**Keywords:** biological invasion front, cropland connectivity, genetic divergence, landscape genetics, least‐cost path

## Abstract

The population genetic structure of invasive species can be strongly affected by environmental and landscape barriers to dispersal. Disentangling the relative contributions of these factors to genetic divergence among invading populations is a fundamental goal of landscape genetics with important implications for invasion management. Here, we relate patterns of genetic divergence in a global invasive agricultural pest, Colorado potato beetle (CPB; *Leptinotarsa decemlineata*), to environmental and landscape factors along an invasion front in Northwestern China. We first used microsatellite markers and spatial‐temporal samples to assess broad patterns of genetic diversity as well as fine‐scale changes in patterns of genetic divergence. We then distinguished the relative contributions of five factors to genetic divergence among front populations: geographic distance (isolation by distance), climate dissimilarity (isolation by environment), and least‐cost distances (isolation by resistance) modeled with three factors: climate suitability, cropland cover, and road networks. Genetic diversity broadly decreased from West to East, with the exception being Eastern China. Low levels of genetic diversity and varying degrees of divergence were observed in Northwestern China, reflecting the potential effect of landscape heterogeneity. Least‐cost distance across cropland cover was most positively correlated with genetic divergence, suggesting a role of croplands in facilitating gene flow. The contribution of climate to genetic divergence was secondary, whether modeled in terms of local adaptability or connectivity of the climatic landscape, suggesting that constraints to CPB gene flow imposed by a harsh climate may be ameliorated in agricultural landscapes. No evidence was found for an obvious effect of road networks on genetic divergence and population structuring. Our study provides an example of how agricultural landscape connectivity can facilitate the spread of invasive pests, even across a broad climatic gradient. More broadly, our findings can guide decisions about future land management for mitigating further spread.

## 1 INTRODUCTION

In this era of globalization, many species have expanded their ranges to the detriment of local communities and ecosystems (Kenis et al., 2009; Simberloff et al., 2013). Successful invasion often follows the breaching of some physical barrier(s) to dispersal and can be accompanied by adaptation to local environmental conditions (Andrade‐Restrepo et al., 2019; Novak, 2007). Environmental and landscape features along an invasion front can therefore strongly influence the distribution of genetic variation among invasive populations (Renault et al., 2018). Central to the field of landscape genetics is elucidation of these underlying processes shaping genetic structure and gene flow, which can be a key step toward developing a long‐term approach for managing and preventing further invasive expansion (Epps & Keyghobadi, 2015; Manel et al., 2003).

Multiple processes may affect the spatial distribution of genetic variation, such as isolation by distance (IBD), isolation by environment (IBE), and isolation by resistance (IBR). IBD, or the phenomenon in which stepwise patterns of gene flow result in greater genetic divergence as the geographic distance between populations increases, can serve as a null model when testing for environmental and landscape effects on genetic divergence (Pelletier & Carstens, 2018; Wang et al., 2012; Wright, 1943). IBE can increase genetic divergence by limiting dispersal or reducing hybrid fitness in heterogeneous environments (Edelaar & Bolnick, 2012; Wang & Bradburd, 2014). IBR (Adriaensen et al., 2003; McRae, 2006) can increase genetic divergence through the effects of landscape features (e.g., topography, land cover composition) on dispersal, and could be especially influential for invasive species undergoing continuous range expansion (O'Reilly‐Nugent et al., 2016).

Numerous studies on invasive species have found evidence of strong effects of environmental and landscape factors on patterns of genetic divergence (Bélouard et al., 2019; Cao et al., 2016; Hoffmann, 2017; Williams et al., 2016). However, the suitability of IBD, IBE, and IBR for explaining patterns of genetic variation varies among species (Sexton et al., 2014) and can be difficult to distinguish when environmental and landscape variables are highly correlated across space. In addition, dispersal and range expansions of agricultural pests can be facilitated by human transportation networks and trade (Heather & Hallman, 2008), helping invasive populations traverse otherwise impassible physical barriers. Therefore, in some cases, we may only vaguely understand the true drivers for genetic divergence among invasive populations.

The now‐globally distributed Colorado potato beetle (CPB), *Leptinotarsa decemlineata* Say (Coleoptera: Chrysomelidae), is a quarantine agricultural pest native to North America that invaded Europe in the early 1900s (de Wilde & Hsiao, 1981; Hurst, 1975), experiencing a strong bottleneck effect (Grapputo et al., 2005). CPB subsequently spread eastward through Central Asia (Weber, 2003), reaching Xinjiang, China, by 1993 (Liu et al., 2012). In Xinjiang, three distinct invasions were detected, which have given rise to isolated CPB populations in three distinct regions within the Gurbantunggut Desert and Tianshan Mountains (Liu et al., 2012; Yang, Guo, et al., 2020; Zhang et al., 2013). The well‐known invasion history of CPB and the diversity of environmental and landscape features along its invasion front offer a unique opportunity to examine which features have had an important influence on CPB gene flow and genetic divergence.

Rapid evolution can accompany invasions and population expansions, but is often missed by landscape genetics studies that focus sampling effort on a single point in time (Hoffmann, 2017; Messer et al., 2016; Prentis et al., 2008; Schwartz et al., 2007). Rapid changes in population structure could have important implications for pest management, for example, when invasive pests have demonstrated an ability to rapidly adapt to management practices (e.g., insecticide use) in the invasive range (Cingel et al., 2016). Including a temporal perspective in assessments of population structure in invasive species can also help identify multiple introductions that might otherwise go undetected (Schwartz et al., 2007).

In this study, we examined patterns of genetic diversity among CPB populations along an invasion front, and identified landscape and environmental factors that have influenced genetic divergence among populations. Our first objective was to quantify genetic diversity and population structure across multiple spatial and temporal scales using a set of nine microsatellite markers. We expected to see reductions in genetic diversity and greater divergence among populations from West to East along the invasion front. Our second objective was to examine the relative contributions of environmental and landscape factors (IBD, IBE, and IBR) to the genetic divergence observed among CPB populations in the invasion front, which we did using a variety of landscape genetics approaches. In the complex terrain and harsh environment of the invasion front, we hypothesized that geographic distance, habitat (cropland) connectivity, climate (temperature and precipitation), and transportation infrastructure (roads) may affect the genetic divergence between populations, though the magnitude of their impact might differ. Having a greater understanding of how these factors contribute to genetic divergence among CPB populations can guide decisions about future land management practices for mitigating further spread.

## 2 MATERIALS AND METHODS

### 2.1 CPB collection

**FIGURE 1:**
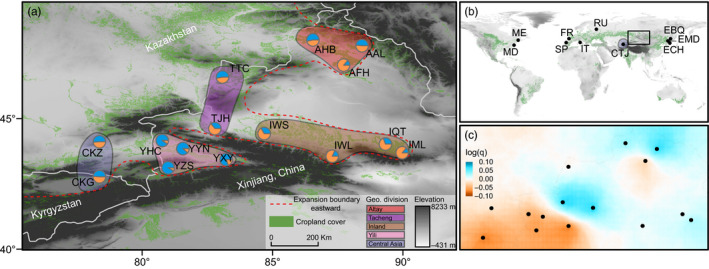
Sample locations of the Colorado potato beetle (a) at the local scale along an invasion front, and (b) at the global scale. The population codes and coordinates are presented in Table S1. Genetic structure of invasion front populations inferred from STRUCTURE analysis at *K* = 2 is shown as pie charts (same as Figure 3b). The color in each pie chart represents the frequency of each cluster in the population. The red dotted line roughly illustrates the boundary of eastward expansion currently observed. Adjacent populations are divided into different geographic divisions and are represented by five different background colors; (c) estimated effective migration surface (posterior mean migration rates on a log_10_ scale) in the invasion front. Colors from cyan to orange indicate high and low effective migration rates, respectively. The black dots indicate the sampled populations, which is consistent with (a)

We opportunistically collected 1,068 CPB adults by hand from commercial potato fields at 15 locations near Xinjiang between 2003 and 2018 (Figure ; Table S1), eight of which were sampled in multiple years. The 15 sample locations can be divided into five geographic divisions: Central Asia, Yili, Altay, Tacheng, Inland (Figure 1a; Table S1), abbreviated respectively as C, Y, A, T, I.

To enable broader geographic comparisons, we collected 236 CPB individuals from 10 populations in USA, Europe, and Eastern China between 2010 and 2016 (Figure 1b; Table S1), in which 60 individuals in Eastern China were from a previous study (Yang, Guo, et al., 2020). After 2016, CPB was never found in Eastern China, which is why we still call the nearby Northwestern China the invasion front.

In total, we sampled 1,304 individuals from 47 populations representing 25 locations. All individuals were killed with 75% ethanol and then stored in 95% ethanol at −40°C until DNA extraction.

### 2.2 DNA isolation, microsatellite genotyping, and genetic diversity

Genomic DNA was extracted from all six legs of each beetle (or from the head, when extracting from larvae) with a DNeasy Blood & Tissue Kit (Qiagen) following the manufacturer's protocol. Nine microsatellite loci for CPB developed and characterized by Grapputo (2006; LdAC5‐2, LdA11b, LdE11c, LdE10e, LdB8b, LdGA4‐18, LdAC5‐22, LdGA4‐5, and LdGA5‐30) were used for genotyping. PCR amplifications followed the methods of Grapputo (2006) with adjusted annealing temperatures set to 52°C, 52°C, 55°C, 55°C, 50°C, 50°C, 47°C, 50°C and 50°C, respectively. Amplified products were sized on an ABI 3730xl DNA Analyzer (Applied Biosystems) by Sangon Biotechnology Co. Ltd. Genotypes were determined using Gene Marker v.1.85 Demo (SoftGenetics). To ensure data quality, we checked for stuttering and large allele dropout using MICRO‐CHECKER 2.2.3 (van Oosterhout et al., 2004). Null allele frequencies were checked using FreeNA (Chapuis & Estoup, 2007) with the EM algorithm (Dempster et al., 1977).

We tested for Hardy–Weinberg equilibrium at each locus at each site and for linkage disequilibrium between each pair of loci using GENEPOP 4.2.1 (Rousset, 2008). Genetic diversity for each population was described using unbiased gene diversity (*H*s), observed heterozygosity (*H*_O_), inbreeding coefficient (*F*_IS_), allelic richness (*A*_R_), and population‐specific *F*_ST_, *β*s (Weir & Goudet, 2017), using the *hierfstat* R package (Goudet, 2004). *A*_R_ was calculated with a rarefaction approach based on the minimum sample size of 12 individuals. These parameters were calculated at the population level and summed across broader geographic divisions, which included: USA, Europe, Central Asia, Yili, Tacheng, Altay, Inland, and Eastern China.

### 2.3 Global and local population structure

To better contextualize fine‐scale patterns of genetic structure along the invasion front, we quantified the degree of genetic divergence among populations from USA, Europe, five regions in invasion front, and Eastern China using pairwise *F*_ST_ and *Gʺ*_ST_, calculated in GenAlEx 6.5 (Peakall & Smouse, 2005), testing for significance with 999 permutations.

We also quantified population structure at global and local scales using two approaches. First, we examined patterns of admixture at a global scale using the Bayesian model‐based cluster analysis implemented in STRUCTURE 2.3.4 (Pritchard et al., 2000). An admixture model with correlated allele frequencies was chosen without considering prior information about geographic proximity. Twenty independent runs were performed for *K* = 1–10, with a burn‐in period of 200,000 iterations, followed by 1,000,000 Markov Chain Monte Carlo iterations. The optimal *K* value was determined using the Δ*K* method (Evanno et al., 2005) implemented in STRUCTURE HARVESTER (Earl & vonHoldt, 2012). Merging of the replicated runs at the optimal *K* value was done in CLUMPP (Jakobsson & Rosenberg, 2007), and results were visualized using DISTRUCT (Rosenberg, 2003). To characterize fine‐scale patterns of CPB population structure in the invasion front, we repeated the STRUCTURE analysis with the same parameters, but this time limiting the analysis to populations from Central Asia, Yili, Altay, Tacheng, and Inland (Figure 1a). For the second approach, we explored clustering among CPB individuals using discriminant analysis of principal components (DAPC), implemented with the *adegenet* R package (Jombart, 2008). Unlike STRUCTURE, DAPC does not rely on any population genetic model assumptions, such as Hardy–Weinberg equilibrium, that are unlikely to be realized among outbreaking populations (Whitlock, 1992). All principal components transformed from the microsatellite data were submitted to a linear discriminant analysis with the first two axes retained. We repeated the analysis excluding populations from USA and Eastern China to enhance visualization of fine‐scale patterns of population structure among populations from Europe and Northwestern China.

### 2.4 Temporal change in population structure

Changes in genetic structure over time were assessed for eight populations (YHC, YZS, AHB, AAL, TTC, IWS, IWL, and IML; Figure 1) sampled in successive years between 2003 and 2018. We performed a principal coordinate analysis in GenAlEx 6.5 (Peakall & Smouse, 2005) with all temporal collections based on a Codom‐Genotypic genetic distance matrix, and visualized temporal variation by connecting points of same locations in chronological order with smooth lines. To investigate the relative extent of spatial and temporal variation, we conducted a permutational multivariate analysis of variance using the *adonis* function in the *vegan* R package (Oksanen et al., 2019). This function provides an alternative to AMOVA (nested analysis of molecular variance; Excoffier et al., 1992) for both crossed and nested factors. We tested the effects of site and year as crossed factors on the individual genetic distance matrix. The individual Euclidean genetic distance matrix was calculated using the *adegenet* R package.

### 2.5 Genetic, landscape, and environmental distances

The 15 populations at the invasion front (Figure 1a) were used for subsequent landscape genetics analyses (genetic distance calculation), in which the multiple year samples at the same location were combined as one population. Genetic distance was estimated as *F*_ST_/(1 − *F*_ST_) in which values of pairwise *F*_ST_ between populations were calculated using GENEPOP (Rousset, 2008). We also repeated all analyses with Nei's *D* genetic distance (Nei, 1972). For distance‐based redundancy analysis (dbRDA, described in Section 2.6), we calculated the individual genetic distance using the *dist* function in R based on allele frequencies that were obtained using the *tab* function in the *adegenet* R package.

Pairwise geographic distances among sampling localities along the invasive front were calculated using the *pointDistance* function in the *raster* R package (Hijmans, 2020). IBE was represented as climatic dissimilarity, estimated using the WorldClim's 19 bioclimatic variables (Fick & Hijmans, 2017). Briefly, these variables represent annual trends, seasonality, and extreme or limiting environmental factors derived from global, monthly temperature and rainfall observations. We extracted the climate variables based on sample site coordinates using the *raster* R package. To reduce collinearity, we performed a principal component analysis (PCA) on the extracted climate variables among sample sites using the *ade4* R package (Dray & Dufour, 2007) and retained the first two axes for calculation of Euclidean environmental distance (using the *dist* function in R). The first two axes of PCA were also used as environmental predictors in following generalized dissimilarity modeling (GDM) analysis.

To quantify landscape resistance, we computed least‐cost distances (LCDs) based on three factors hypothesized to effect CPB dispersal and gene flow: (a) climate suitability (IBR‐climate); (b) cropland cover (IBR‐landcover); and (c) road networks (IBR‐road).

Least‐cost distances for the three landscape resistance surfaces (LCD‐climate, LCD‐landcover, and LCD‐road) were calculated among all pairwise population combinations using the *costDistance* function in the *gdistance* R package (van Etten, 2017). Under this framework, grid values range from 0–1, with 1 corresponding to no landscape resistance. We also used the *shortestPath* function to visualize least‐cost paths on the three surfaces.

All inputs for the subsequent tests (partial Mantel test, GDM, and dbRDA) of isolation models (IBD, IBE, and IBRs) are summarized in Table S2.

#### 2.5.1 Isolation by resistance‐climate

A climate suitability surface that encompassed the invasion front region (74°E–91°E, 39°N–51°N) was constructed using an ecological niche model (ENM) implemented with MaxEnt 3.4.1 (Phillips et al., 2006), with 15 replicates of subsampling, and reserving 15% of samples as the training data set for model evaluation. 19 bioclimatic variables related to temperature and precipitation were obtained from Worldclim.org (Fick & Hijmans, 2017), and all were retained as environmental predictors to build the ENM, without filtering collinear variables because the regularization algorithm in MaxEnt accounts for collinearity in predictors (Elith et al., 2011). We obtained CPB distribution records from online databases (GBIF, CABI, EPPO). However, due to the paucity of records of CPB in Asia, we also included distribution data from surveys conducted by our research team in Kazakhstan, Kyrgyzstan and Xinjiang, China. A jackknife test implemented with MaxEnt was used to test the contribution of each variable, and the area under the receiver operating characteristic curve (AUC) value was used for model evaluation.

#### 2.5.2 Isolation by resistance‐landcover

We expected that cropland cover connectivity plays an important role in CPB dispersal and gene flow, as CPB is almost exclusively observed in commercial potato fields, and the distribution of wild hosts (e.g., *Solanum rostratum*) is highly restricted (Wang et al., 2017). To model the effects of cropland cover connectivity, we created a second ENM with the same settings as the previous one, but also included land cover classification as a categorical environmental predictor. We obtained the global land cover layer (Tateishi et al., 2014), which contains 20 land cover categories corresponding to types of natural, urban, and cropland cover, where the croplands category includes land cover for all herbaceous crops (e.g., potato). Prior to ENM construction with MaxEnt, we resampled the global land cover layer to 30 arc‐second resolution using ArcGIS 10.2.2.

#### 2.5.3 Isolation by resistance‐road

We included a resistance surface based on the configuration of major roads because we expected that CPB might be dispersed by movement of host material along roadways (Li, Cheng, Guo, et al., 2013), regardless of the presence of any geographic barriers. We obtained data on major roads from Natural Earth Data (https://www.naturalearthdata.com/), and created a raster layer where roads were designated as 1 and nonroads as 0 in ArcGIS 10.2.2. We matched CPB populations to the road resistance surface by adjusting population coordinates to the nearest road.

### 2.6 Landscape genetics analyses

We used estimated effective migration surfaces (EEMS; Petkova et al., 2016) to visualize genetic divergence among CPB populations over the landscape. EEMS estimates effective migration rates from observed genetic distances among population samples, then interpolates values of effective migration over a spatial extent. We ran EEMS using 1,000 demes, with two independent starting chains for 2,000,000 MCMC iterations following a burn‐in of 500,000, with a thinning of 10,000.

As an initial exploration of IBD, IBE, and IBRs, we fit a linear model using multiple matrix regression with randomization (MMRR; Wang, 2013). We performed the MMRR test of genetic distance with geographic distance, environmental distance and LCDs, respectively, with 999 permutations.

We used three statistical methods to determine the relative importance of climate and landscape variables on genetic divergence: (a) partial Mantel test using the *vegan* R package; (b) GDM (Ferrier et al., 2007) using the *gdm* R package (Fitzpatrick et al., 2020); and (c) dbRDA (McArdle & Anderson, 2001) using the *vegan* R package.

Partial Mantel test improve upon Mantel test by allowing the examination of the correlation between genetic distance matrix and some environmental distance matrix (e.g., climate or landscape resistance distances), after accounting for any correlations with another variable (often geographic distance matrix). GDM is a matrix regression technique, that calculates the relative (IBD, IBE, IBRs) and combined (IBD + IBE, IBE + IBR‐landcover, All) contributions of predictor variables to genetic divergence. dbRDA is a constrained linear ordination method that combines multiple linear regression and principle component analysis. We used dbRDA to detect whether there are some environmental factors promoting genetic divergence, after having removed the distance‐based effect by constraining the distance variable with the highest degree interpretation to genetic divergence (i.e., LCD‐landcover distance, indicated by GDM model). The environment variables that we considered were the 19 bioclimatic factors extracted from individual coordinates. To avoid high collinearity of all variables, we performed loop pre‐dbRDAs, removing the variable with the highest variance inflation factor (VIF) each run, until VIF values of the remaining variables were <10. The following variables were retained: Bio1 (annual mean temperature), Bio2 (mean diurnal range), Bio6 (minimum temperature of the coldest month), Bio8 (mean temperature of the wettest quarter), Bio12 (annual precipitation), and Bio15 (precipitation seasonality). Pairwise LCD‐landcover distance at the population level was allocated to the individual pairs and then transformed into principal coordinates of neighborhood matrices (PCNMs) as rectangular data. We tested the respective and relative effect of environment variables on genetic divergence according to two models: a model with only climate variables, and a model with climate variables controlled by PCNM variables. For comparability, we also tested two other models: a model with only PCNM variables, and a model with PCNM variables controlled by climate variables.

## 3 RESULTS

### 3.1 Global genetic diversity and population structure

A heterozygote deficit was detected in 25 of the 423 locus‐population pairs, while 24 pairs showed heterozygote excess after Bonferroni correction (*p* < .01). Linkage disequilibrium was observed for 33 of the 1,692 locus‐locus pairs within populations and in two of 36 pairs across all populations (*p* < .01). Importantly, no locus exhibited significant deviations from Hardy–Weinberg or evidence of linkage disequilibrium across all populations.

**FIGURE 2:**
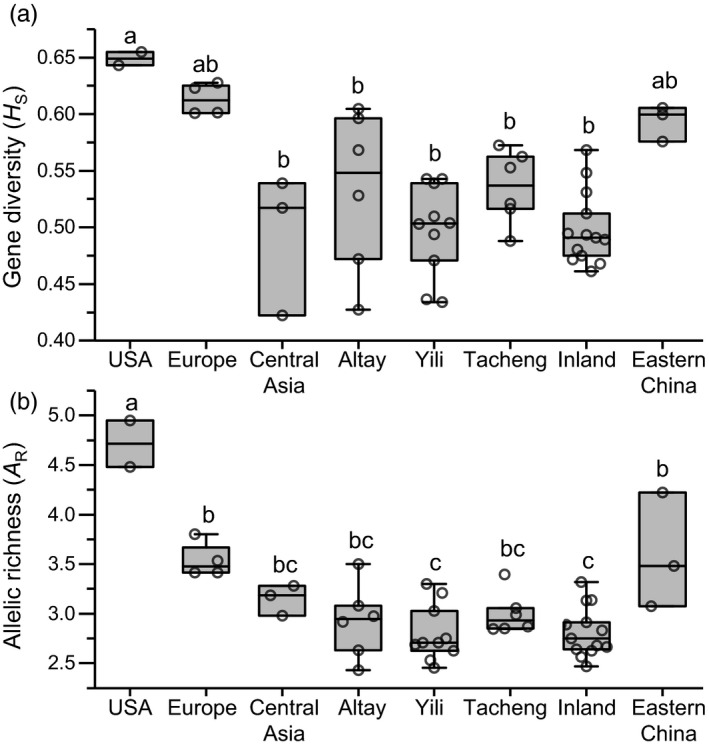
Genetic diversity of Colorado potato beetle populations among geographic divisions, according to (a) gene diversity (*H*_S_) and (b) rarefied allelic richness (*A*_R_). Raw data points are shown together with boxplots and the values for each population are available in Table S1. Letters denote significant differences between geographic divisions (*p* < .05 after Bonferroni correction). Geographic divisions are the same as in Figure 1 and Table S1

USA populations exhibited the highest genetic diversity (across all metrics), followed by Europe then Asia. Gene diversity (*H*_S_) ranged from 0.643 to 0.655 in the USA, from 0.601 to 0.628 in Europe, from 0.422 to 0.539 in Central Asia, and from 0.433 to 0.608 in Xinjiang, China (Figure 2a; Table S1). Unexpectedly, populations in Eastern China had the highest levels of genetic diversity in Asia, ranging from 0.576 to 0.600. Allelic richness showed a similar pattern (Figure 2b). Most Asia populations were highly genetically differentiated from populations in Europe (*β*s > 0.05), except for populations in Altay and Eastern China (0.020 to −0.002; Table S1).

**FIGURE 3:**
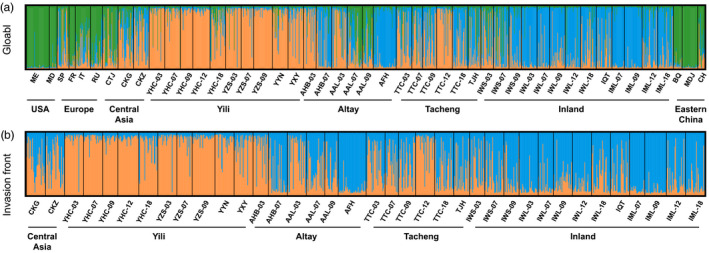
Barplots of STRUCTURE clustering analyses for Colorado potato beetle populations at the (a) global scale (*K* = 3) and (b) local scale along the invasion front (*K* = 2). Each individual is denoted by a narrow vertical bar and its proportional membership in each of *K* cluster is represented by a different color. Geographic divisions are the same as in Figure 1

Pairwise *F*_ST_ among the eight regions ranged from 0.008 to 0.118, and *G’’*_ST_ ranged from 0.029 to 0.511 (Table 1). Global STRUCTURE analysis identified three clusters (Figure S1a), two of which encompassed populations in the invasion front (Figure 3). DAPC analysis identified populations from the USA and Eastern China as highly divergent from each other and from European and other Asian populations (Figure S2).

**TABLE 1:** Pairwise *F*_ST_ (lower left) and *G*ʺ_ST_ (upper right) of Colorado potato beetle populations between the eight sample regions

	USA	Europe	Yili	Altay	Tacheng	Inland	Central Asia	Eastern China
USA		0.406	0.511	0.426	0.476	0.483	0.489	0.394
Europe	0.079		0.207	0.079	0.139	0.205	0.173	0.248
Yili	0.118	0.047		0.118	0.070	0.226	0.180	0.327
Altay	0.092	0.018	0.028		0.029	0.065	0.116	0.277
Tacheng	0.106	0.031	0.018	0.008		0.091	0.119	0.314
Inland	0.112	0.046	0.057	0.016	0.023		0.200	0.370
Central Asia	0.113	0.040	0.046	0.029	0.030	0.052		0.257
Eastern China	0.077	0.049	0.074	0.060	0.069	0.085	0.059	

### 3.2 Spatial‐temporal genetic divergence among front populations

Two clusters were identified in the fine‐scale STRUCTURE analysis (Figure 3b; Figure S1b), which showed different clustering patterns in different areas along the invasion front. Populations from Yili and Inland were clearly assigned to distinct clusters, while populations along the western and northern borders of China exhibited admixture (Figure 1; Figure S1). DAPC analysis showed separation of the European populations (RU, FR, and IT), as well as modest divergence among populations along the invasion front (Figure S2b).

Our analysis of change in population structure over time revealed substantial changes in TTC in Tacheng, and AAL and AHB in Altay (Figure 3; Figure S3). According to the permutation‐based multivariate analysis of variance, we found a significant effect of site and year (Table S3). However, the interpretation by site (*R* ^2^ = .09) was much greater than year (*R* ^2^ = .02), although several sites were not completely symmetrical in temporal collection.

### 3.3 Ecological niche modeling for resistance surfaces

**FIGURE 4:**
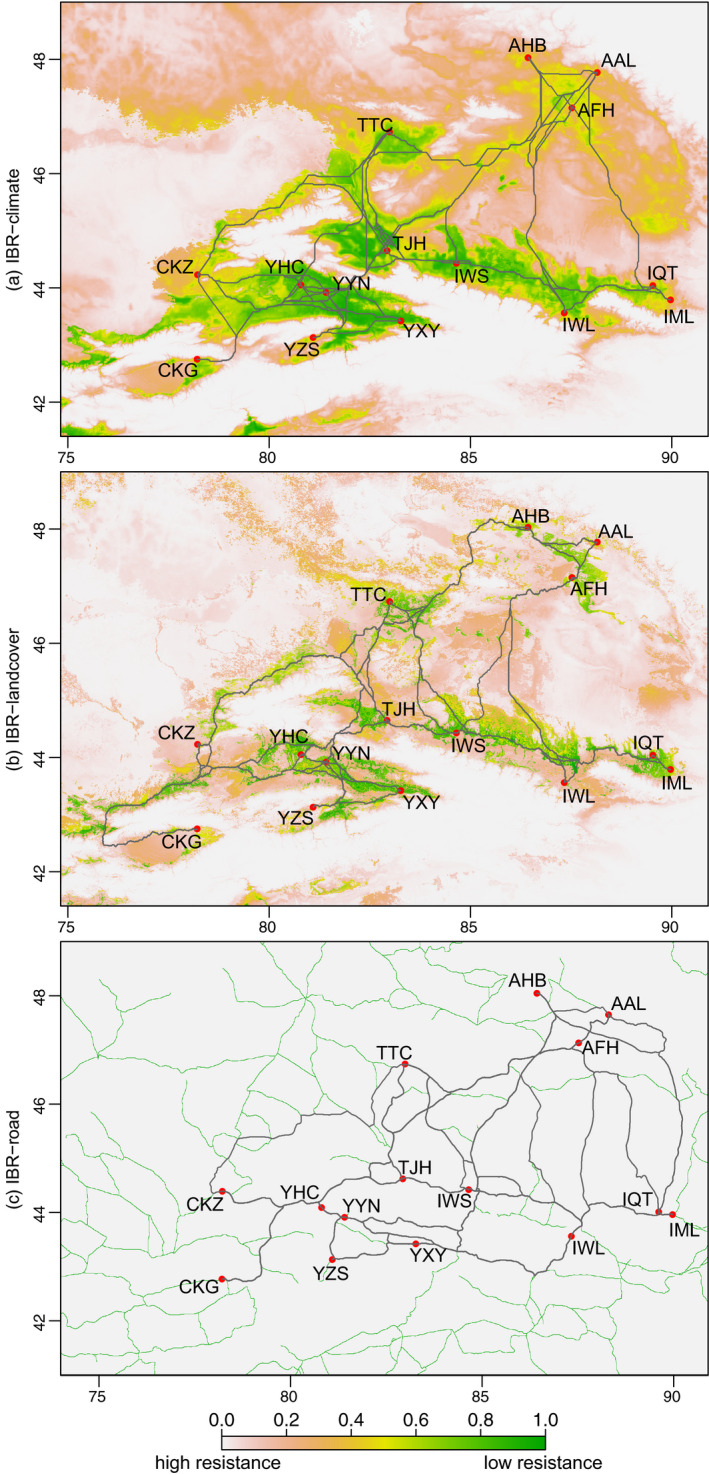
Landscape resistance surfaces and least‐cost paths used for landscape genetics analysis. (a) isolation by resistance (IBR)‐climate, (b) IBR‐landcover, and (c) IBR‐road. Regions with lower values indicate putatively higher resistances to beetle movement. Population locations are shown as red dots. The gray line between populations depicts the least‐cost path

The two ENMs used to create landscape resistance surfaces showed credible performance (AUC = 0.99). The jackknife tests indicated that two variables: Bio1 (annual mean temperature) and Bio18 (precipitation of warmest quarter) contributed the most to isolation in the first ENM (Figure S3a), and land cover contributed the most to isolation, along with Bio1 (annual mean temperature) in the second ENM (Figure S4b). Among land cover predictors, cropland cover and urban land cover, which we respectively expect to facilitate or impede gene flow, had greater contributions than did other landcover types (Figure S4c). The climate and land cover ENMs suggested that the most suitable habitat and least‐cost paths for CPB occur along the foothills and valleys of the Tianshan mountains (Figure 4).

### 3.4 Landscape and environmental effects on genetic divergence

Visualization with EEMS highlighted regions with lower and higher effective migration rates than IBD expectations (Figure 1c). High migration rates occurred in Yili, and between Tacheng to Inland, while low migration rates were visible along the mountains and Gurbantonggut Desert (areas below population AFH).

MMRR tests of IBD and IBRs initially showed extremely significant results with *p* values ranging from .0004 to .0009, *R* ^2^ from .131 to .237, and with the IBR‐landcover model showing the highest correlation. The MMRR test result for IBE was also significant (*p* = .0323, *R* ^2^ = .116; Table 2).

**TABLE 2:** Results of multiple matrix regression with randomization (MMRR), partial Mantel test, and generalized dissimilarity modeling (GDM) to evaluate the isolated and combined contributions of geographic distance, environment distance and three least‐cost distances on Colorado potato beetle population genetic divergence (measured as *F*_ST_/(1 − *F*_ST_))

Models	MMRR		Partial Mantel test		Proportion genetic divergence explained in GDM (%)
	*R^2^*	*p*	*r*	*p*	
IBD	0.131	9E−04	0.23	0.039	14.10
IBE	0.116	0.032	0.188	0.141	26.00
IBR‐climate	0.148	0.005	0.282	0.036	19.89
IBR‐landcover	0.237	4E−04	0.449	0.006	29.66
IBR‐road	0.138	7E−04	0.128	0.207	15.26
IBD + IBE					31.38
IBR‐landcover + IBE					38.81
All					38.82

Abbreviations: IBD, isolation by distance; IBE, isolation by environment; IBR, isolation by resistance.

Results of partial Mantel tests and GDM are presented in Table 2. Only IBR‐landcover remained significant at *p* < .01 after constraining for the effects of geographic distance (*p* = .006, *r* = .449). In GDM models, all predictor variables (All; IBD + IBE + IBRs) together explained 38.82% of genetic divergence among CPB populations. IBR‐landcover was the most important explanatory variable, explaining 29.66%, and IBE independently explained 26.00% of the observed genetic divergence. IBR‐landcover and IBE together explained 38.81%. Similar results were obtained when using Nei's *D* as genetic distance (Table S4).

**FIGURE 5:**
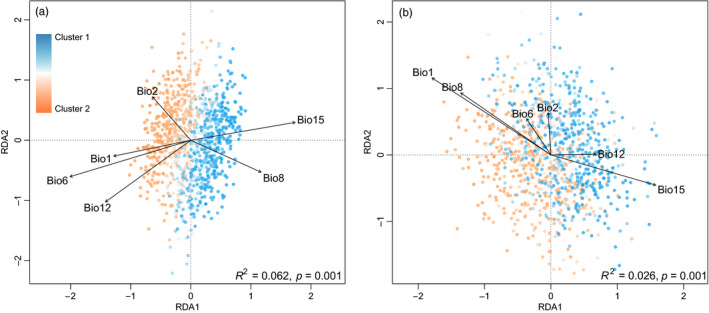
Distance‐based redundancy analyses (dbRDAs) on individual genetic distance explained by the environmental effects of climate. (a) dbRDA model considering only climatic effects, and (b) partial dbRDA model constraining least‐cost distance‐landcover effect to analyze the independent effect of climatic variables. Bio1, annual mean temperature; Bio2, mean diurnal range; Bio6, minimum temperature of coldest month; Bio8, mean temperature of wettest quarter; Bio12, annual precipitation; Bio15, precipitation seasonality. Individuals were colored according to the proportion assigned to the two STRUCTURE clusters (Figure 3b). Longer arrows indicate higher correlations between variable and genetic distance in the corresponding direction

Distance‐based redundancy analysis analyses also identified several climate variables that significantly predicted genetic divergence (*R* ^2^ = .062, *p* = .001 for climate only; Figure 5a; *R* ^2^ = .026, *p* = .001 for LCD‐landcover controlled; Figure 5b). The climate variables together explained only 3.08% of genetic variation after accounting for effects of LCD‐landcover (Figure 5b), in which Bio12 (annual precipitation) and Bio15 (precipitation seasonality) were highly positively related to most individuals from the Inland region (the blue cluster, Figure 3b). When using LCD‐landcover as the explanatory variable and climate variables as the control variables, landcover explained 6.37% of the genetic variance.

## 4 DISCUSSION

### 4.1 Relationship between Asian invasion and agricultural landscapes

Our study revealed a clear geographic pattern of genetic diversity and population structure at global as well as local scales (Figures 1, 2, 3). CPB populations from United States and Europe had relatively high genetic diversity, while populations in Western China had very low genetic diversity. This pattern is not only similar to Grapputo et al. (2005), supporting a rapid expansion throughout Europe after a single introduction from the United States, but also generally consistent with the stepwise expansion history of CPB in Europe and Asia (Boiteau et al., 2003; Liu et al., 2012). One surprising finding was high levels of genetic diversity among CPB populations in Eastern China as well as evidence of admixture with (or more recent divergence from) European lineages (Figure S1a).

Overall, our findings suggest that CPB populations are diverging along the invasion front in Asia, largely driven by low connectivity of cropland cover. This suggests that croplands in Northwestern China provide opportunities for the rapid spread of CPB, in a pattern consistent with experimental and observational studies in North America (Boiteau et al., 2003; Crossley et al., 2019a, 2019b; Voss & Ferro, 1990). Although populations near Western China were recorded to have all originated from Europe at similar times around 1979–1993 (Jolivet, 1991; Liu et al., 2012; de Wilde & Hsiao, 1981), they have shown highly variable levels of genetic divergence (Table 1). Sampling across several years in the Altay region revealed rapid changes in genetic diversity and population structure: The allelic compositions of AHB and AAL changed substantially since 2007 (Figure 3; Figure S3). Interestingly, the AAL‐07 and AAL‐09 populations exhibited high proportions of European ancestry (Figure 3; Figure S1), and had relatively high genetic diversity (Table S1), suggesting that Northeast Kazakhstan and Northwestern Xinjiang could be experiencing secondary contact and admixture between invasive lineages from Europe (Russia) and Central Asia. This is consistent with the idea that the agriculturally intensive regions in Southern Russia and Northwestern Xinjiang offer few physical barriers to CPB dispersal, as has also been demonstrated for other highly mobile invasive species: For example, American minks (Huang et al., 2017), Migratory locust (Ma et al., 2012) and Russian wheat aphid (Zhang et al., 1999), and secondary contact through this agricultural pathway improved genetic diversity of AAL‐07 and AAL‐09 populations in Altay. Importantly, our finding of high genetic diversity among populations within Eastern China demonstrates how genetic diversity can be maintained after a long‐distance expansion, possibly enabled by high agricultural landscape connectivity in south Siberia (Bieńkowski & Orlova‐Bienkowskaja, 2018). In contrast, the sparseness of croplands and the arid, mountainous terrain in Western China could explain the lower genetic diversity, higher genetic divergence, and more stable population structure through time among CPB populations along the northwestern invasion front (Figure S3).

Additional modes of dispersal by CPB may effectively increase gene flow among populations. For example, hitchhiking on vehicles may help CPB break through biogeographic barriers, and CPB can fly over long distance when assisted by strong winds (Termier et al., 1988). Our IBR‐road results suggest that transportation networks play a minimal role in facilitating CPB gene flow, and we did not observe an abnormal distribution of genetic structure across biogeographic barriers. Nevertheless, our EEMS analysis did highlight several areas with high effective migration rates compared to pure IBD expectation, such as the area from Tacheng to Inland (between population TJH and IWS, specifically; Figure 1c), where croplands are sparse. Therefore, though wind‐assisted migration may be rare itself (Boiteau et al., 2003) and its effect difficult to distinguish from that of cropland cover, this does not preclude the possibility of CPB spread over areas with low cropland connectivity.

### 4.2 Relative importance of climate and landscape in shaping genetic divergence

Using CPB's invasion front in Northwestern China as a model system to test associations between climate and landscape variables and genetic divergence, we found that least‐cost distances estimated from ENM‐based cropland cover, followed by IBE, accounted for the highest percent of observed genetic divergence. Their combined contribution of 38.81%, which is close to the 38.82% contribution of all predictors, suggests that the other distance‐based variables may be less important. However, after controlling for effects of IBD, only the effect of cropland cover remained significant (cropland cover alone accounted for 29.66% of genetic divergence; Table 2). These results suggest that the irrigated cropland cover of Northwestern China is crucial for maintaining connectivity among CPB populations and facilitating further spread, whether in a suitable climate or not, and that the constraints on dispersal imposed by harsh climate may be ameliorated by the availability of host crops.

Furthermore, our results suggest that in some geographic divisions such as Inland, genetic divergence is still attributable to patterns of IBD (Figure 1c), which may be due to the contiguous croplands on the northern slope of the Tianshan Mountains. CPB is an oligophagous pest with a relatively low propensity for dispersal, being predominantly sessile as larvae on hosts, and preferring walking over flight as adults (Boiteau et al., 2003). Therefore, landscapes planted with large acreages of potatoes could be considered as a continuous bridgehead for CPB spatial expansion. Future research should determine whether the observed negative association between genetic divergence and cropland cover is due to failure of beetles to effectively find host plants in landscapes with sparse potato cover (a passive isolation scenario), or if concentration of limited potato land cover in a small area acts to attract and retain CPB (a proactive stay scenario). Distinguishing between these alternatives can inform which management strategy to employ for slowing CPB spread: concentrating potato production in areas separated by a broad zone of noncrop habitat, or attracting CPB with trap crop for highly targeted eradication efforts.

### 4.3 Limited evidence of IBE

The harsh temperate continental arid and semi‐arid climate at the invasion front provides an opportunity to study the response of population genetic variation in invasive populations to environmental variation. However, results from all three of our statistical approaches show that the role of climate is secondary, whether in terms of climatic suitability (IBE) for local residents or diffusion across climatic resistance surfaces (IBR‐climate), to that of cropland connectivity in shaping genetic divergence among CPB populations. Though we acknowledge that the statistical power of data from nine microsatellite markers to quantify genetic divergence may be limited, we still consider our data informative, as they were able to distinguish interrelated effects of geographic distance, climate dissimilarity, and landscape resistance on genetic divergence. The climate‐related patterns of IBE should be treated as indicators of how environmental variation might affect population process such as the life cycle and genetic drift. We therefore tentatively suggest that CPB may be able to adapt to constraints imposed by the harsh climate in the invasion front in China, though a detailed analysis of genome‐wide genetic markers among geographic populations is needed.

Despite explaining a relatively small amount of genetic divergence among CPB individuals, our dbRDA analyses still identified several potentially influential climate variables related to temperature (e.g., Bio1, Figure 5) and precipitation (e.g., Bio15, Figure 5). These climate differences are indeed observable on the ground: Inland of Xinjiang, China, is dry and hot, while precipitation is relatively higher in the western border. According to laboratory experiments, summer heat over 39°C and annual precipitation lower than 150mm may be highly restrictive for CPB dispersal and persistence in some areas along the invasion front (Li, Cheng, Liu, et al., 2013, Li et al., 2016). However, any apparent effects of climate on CPB distribution might actually be mediated by climate effects on host plants. Specifically, we found that potato exhibit a higher sensitivity to extreme climate than CPB (Aksoy et al., 2015; Hijmans, 2003; Monneveux et al., 2014), suggesting that restricted host plant range is a better measure of potential CPB distribution and spread than climate‐based models (Wang et al., 2017).

### 4.4 Implications for management

Understanding the sources and main pathways of invasive species is essential for accurate risk assessment and management (Sakai et al., 2001). Our results emphasize the importance of agricultural landscape connectivity for CPB invasion. Land‐use decision and management may help reduce the further spread of CPB and other pests.

Specifically, this study and previous studies (Zhang et al., 2013) have shown that populations in Altay and Eastern China have the highest genetic diversity (Table S1) around China and Central Asia (Table S1), and genetic divergence with European populations is relatively low (Figure 3a), which implies that agriculturally intensified regions of Southern Russia and Northeastern Kazakhstan could be an invasion gateway for the Central Asia and China. As CPB is not the only agricultural pest to have invaded China along this route, increased efforts to block and eradicate CPB and other invasive species in Northeastern Kazakhstan are warranted. In northwest Xinjiang near Kazakhstan, such as Tacheng (TTC) and Habahe (AHB), it is recommended that potato cultivation be spatially restricted to prevent the repeated introduction of highly diverse populations. This is especially important for areas between Yili and Inland, which contain populations belonging to highly divergent invasive lineages. Further admixture among these populations may secure CPB's success in its final invasion front (Rius & Darling, 2014).

## Supporting information

Fig S1‐S4

Table S1‐S4

## Data Availability

Microsatellite data, resistance surface layers, and R scripts are available on Dryad (https://doi.org/10.5061/dryad.pg4f4qrmq; Yang, Liu, et al., 2020).
