# Supplementary material for: Cropland connectivity affects genetic divergence of Colorado potato beetle along an invasion front
Source: Evol Appl. 2020 Oct 8;14(2):553–65. doi: 10.1111/eva.13140 (PMC7896701; doi:10.1111/eva.13140)
Supplement: Supplementary file 1 — Fig S1‐S4 [file EVA-14-553-s001.pdf]

Supplementary Figures for “Cropland connectivity affects genetic divergence of Colorado potato beetle along an invasion front”

Fangyuan Yang[1,2], Ning Liu[2], Michael S. Crossley[3], Pengcheng Wang[2,4], Zhuo Ma[2,4], Jianjun Guo[1], Runzhi Zhang[2,4]

[1]Institute of Entomology, Guizhou University, Guiyang, Guizhou, China
[2]Key Laboratory of Zoological Systematics and Evolution, Institute of Zoology, Chinese Academy of Sciences, Beijing, China
[3]Department of Entomology, University of Georgia, Athens, Georgia, USA
[4]University of Chinese Academy of Sciences, Beijing, China

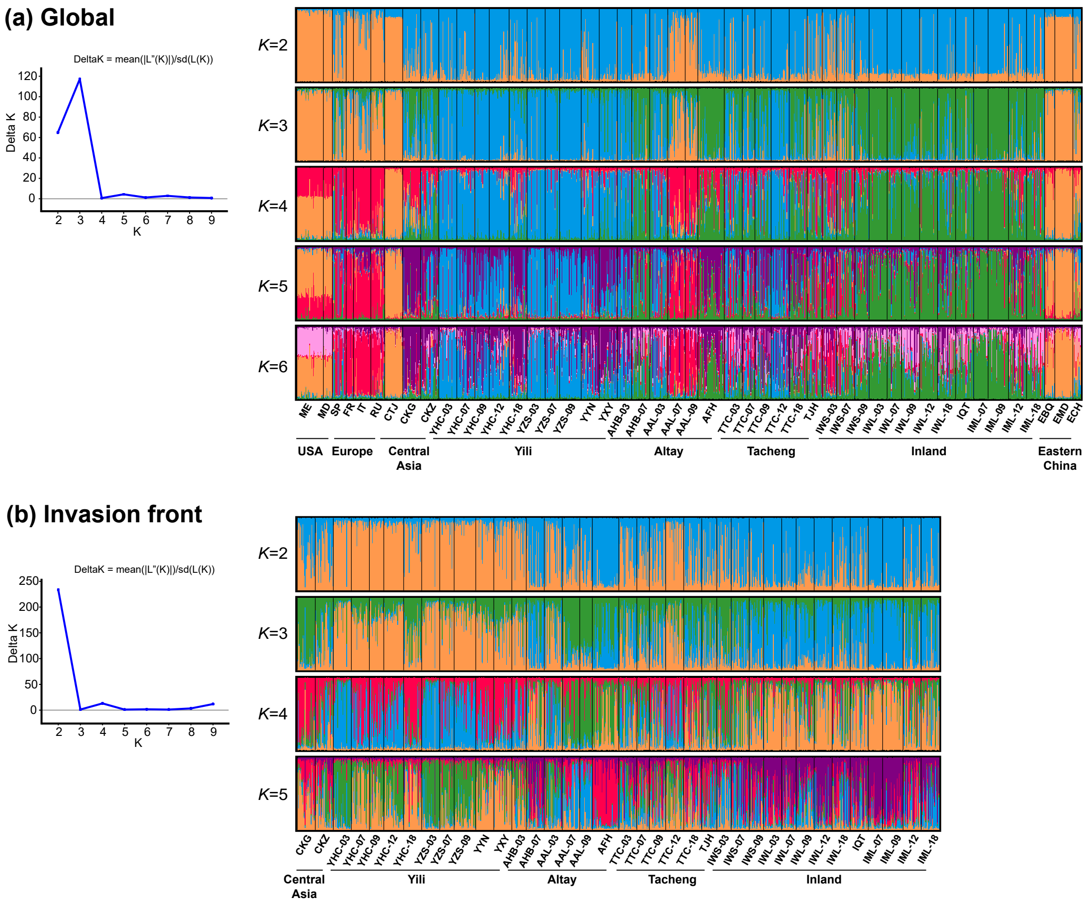

Figure S1 Estimating number of clusters using Δ*K* method with *K* from 2 to 9. Clusters of individuals when *K* at 2-6 are shown for global data set (a), and when *K* at 2-5 are shown for frontier data set (b).

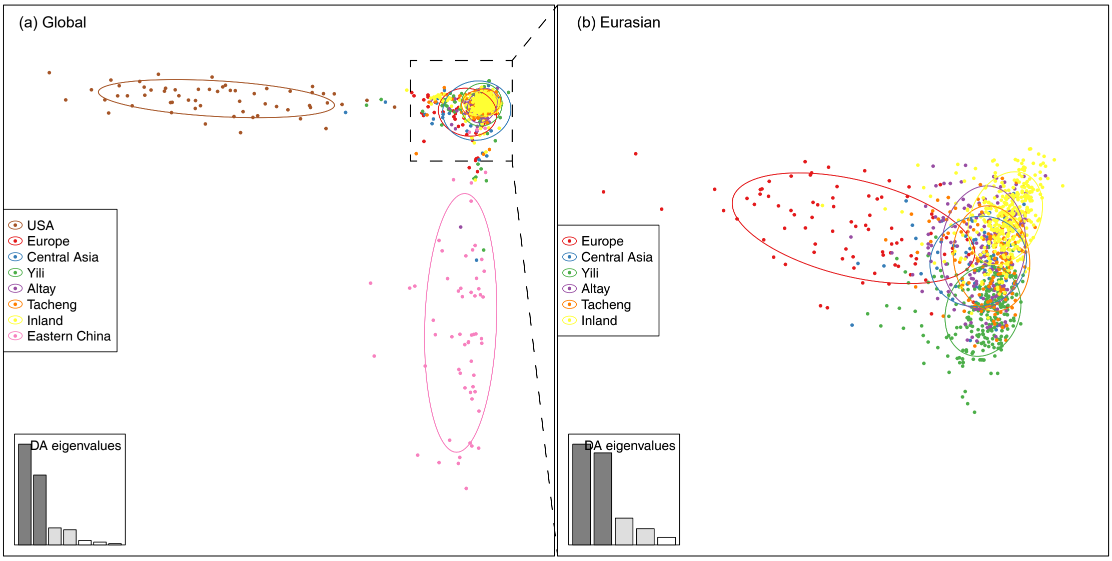

Figure S2 Genetic structure of *Leptinotarsa decemlineata* inferred by two DAPC analyses. The first analysis (a) included all global samples in our study, and the second analysis (b) removed samples from USA and Eastern China.

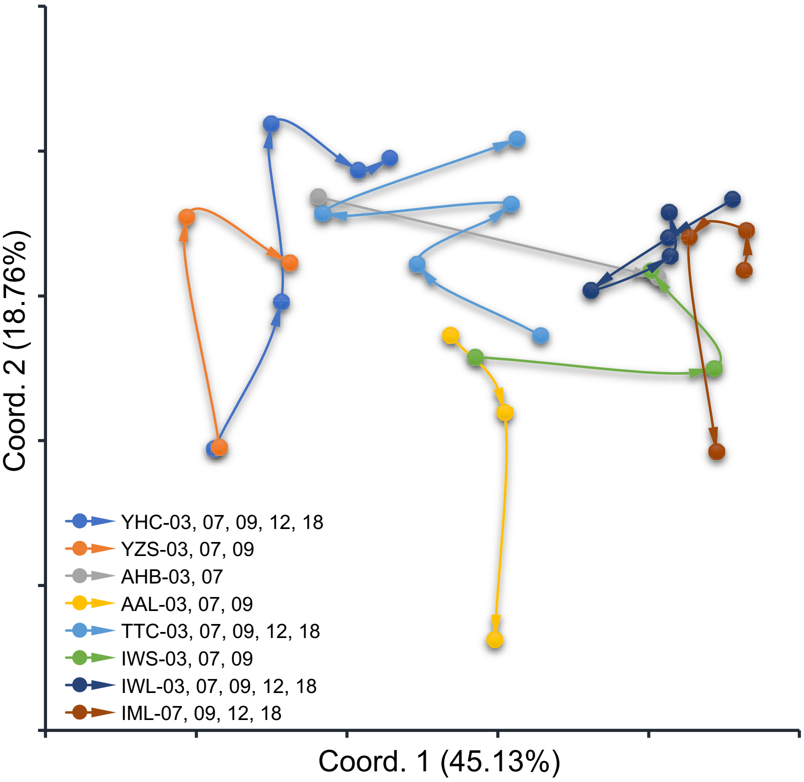

Figure S3 Principal coordinates analysis of pairwise Codom-Genotypic genetic distances calculated for temporal Colorado potato beetle samples at 8 populations in Xinjiang, China. Arrows indicate the direction of change in genetic similarity between years. The percentage variation explained by each principal coordinate (Coord.) is given in brackets. Collection years are indicated next to population codes.

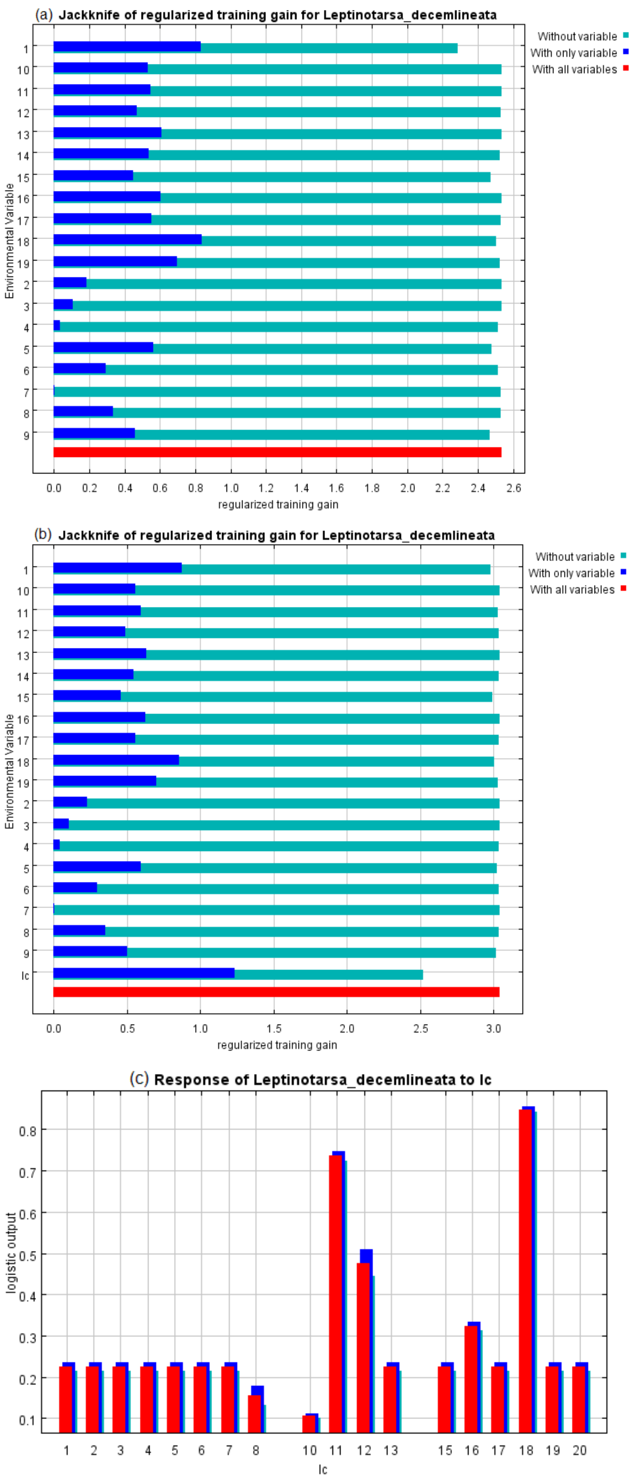

Figure S4 Jackknife test for relative importance of environmental variables in the development of MAXENT models. (a) result of 19 environmental variables for IBR-climate surface; (b) result of 19 environmental variables and land cover categorical variable (lc) for IBR-landcover surface; (c) relative contribution of different land cover types in land cover variable. 11, 12 and 18 refer to cropland cover, paddy field cover and urban cover respectively. For other landcover codes see: https://globalmaps.github.io/glcnmo.html
